# Rheological Theory Applied to Mechanical Ventilation in Acute Respiratory Distress Syndrome: A New Paradigm for Understanding and Preventing Ventilator-Induced Lung Injury

**DOI:** 10.3390/jcm14186544

**Published:** 2025-09-17

**Authors:** Alberto Medina, Pablo del Villar Guerra, Juan Ramón Valle Ortiz, Vicent Modesto I Alapont

**Affiliations:** 1 Paediatric Intensive Care Unit, Hospital Central de Asturias, 33011 Oviedo, Spain; amedinavillanueva@gmail.com; 2 Department of Paediatrics, Hospital El Bierzo Ponferrada, 24404 León, Spain; 3 Cardiac Intensive Care Unit and Paediatric Intensive Care Unit, Newcastle University Hospitals, Newcastle NE7 7DN, UK; juan.valleortiz@nhs.net; 4 Paediatric Intensive Care Unit, Hospital La Fe, 46026 Valencia, Spain; vicent.modesto@gmail.com

**Keywords:** acute respiratory distress syndrome, ventilator-induced lung injury, rheology, mechanical ventilation, driving pressure, mechanical power, strain, stress, lung protective ventilation, materials science

## Abstract

The concept of mechanical power (MP) has emerged as a comprehensive indicator of ventilator-induced lung injury (VILI). It integrates the effects of tidal volume, airway pressures, respiratory rate, and flow. However, applying MP as a universal threshold (e.g., 12 J/min) across heterogeneous patients with acute respiratory distress syndrome (ARDS) may be inadequate. This review introduces the rheological model, which conceptualizes the lung as a viscoelastic body (i.e., one that exhibits both elastic and viscous properties), and applies it to ARDS ventilation. The rheological model may offer individualized MP thresholds. The potential benefits of adjusting MP based on ideal body weight (J/min/kg) are discussed and, more accurately, on static compliance (J/min/L). Static compliance could better reflect functional lung size, though clinical validation remains needed. Preliminary clinical and modeling evidence suggests that normalized MP correlates more closely with mortality than absolute MP and aligns with pulmonary stress–strain behavior. This normalization provides a more precise risk stratification and facilitates the easier setting of ventilation targets, particularly in patients with low compliance or abnormal body composition. This review clarifies definitions and consolidates evidence, highlights the clinical implications of rheology for lung-protective strategies. MP normalization within a lung-protective strategy could enhance the safety and efficacy of mechanical ventilation; however, clinical validation is still required. This review summarizes the theoretical foundations, supporting evidence, and clinical implications of this approach within the broader context of rheological modeling in ARDS.

## 1. Introduction

Acute respiratory distress syndrome (ARDS) represents one of the most significant challenges in critical care. Traditional theories explaining ventilator-induced lung injury (VILI), including barotrauma, volutrauma, atelectrauma, and biotrauma, have shown important limitations in fully capturing the pathophysiological mechanisms of lung damage during mechanical ventilation [1,2].

The application of rheological principles, derived from material science and supported by experimental evidence, offers a new paradigm that may explain VILI mechanisms more comprehensively [3]. Rheology, the study of deformation and flow of matter, considers the lung as a viscoelastic body whose response to mechanical forces depends on their magnitude, frequency, and velocity [4] (Table 1).

Key concepts of rheological theory applied to the lung include the following:

**Stress.** Stress is the value of the maximum limit of force per unit area [4]. It corresponds to the transpulmonary pressure (PTP) difference between inspiration and expiration. PTP is the difference between alveolar pressure (Palv) and pressure within the pleural space (Ppl) [1]. It is assumed that clinically, pleural space pressure (mediastinal pressure) can be estimated by measuring esophageal pressure (Pes).

**Strain, deformation, or relative displacement.** This is the multidimensional deformation suffered by the body, i.e., the change in shape relative to the original state [4]. In classical respiratory physiology, the concept of strain does not exist, but its equivalent could be considered the quotient between tidal volume (V_T_) and functional residual capacity (FRC).

**Figure 1:**
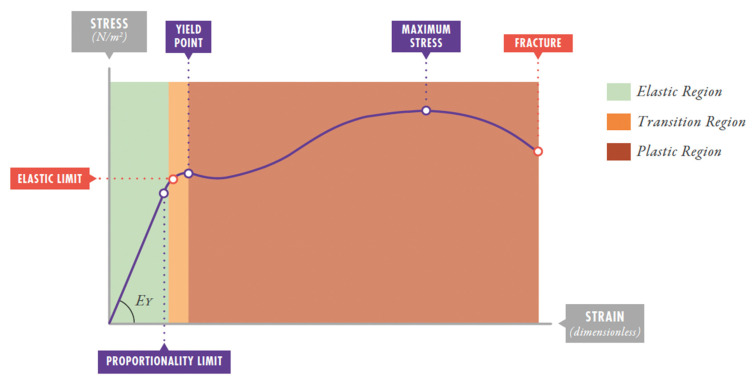
Stress–strain curve. E_Y_: Young’s modulus of elasticity. Reproduction with permission: Manual of Pediatric and Neonatal Mechanical Ventilation (SECIP and ESPNIC). 2nd ed. Medina Villanueva A et al. (Eds.). Las Palmas de Gran Canaria (Spain): Tesela Ediciones. 2021.

**Young’s Modulus (specific lung elastance, Figure 1).** Each material’s behavior is described by what is called the stress–strain curve. In all stress–strain curves, an initial zone that is linear can be identified, defining its elastic behavior [3,5]. The proportionality constant (the slope of this line) E_Y_ (cmH_2_O) is called the Young’s modulus of elasticity of the solid. The Young’s modulus of the respiratory system is known as specific lung elastance (ESL). It acts as the proportionality constant linking stress and strain.

**Strain rate or deformation velocity.** The velocity at which relative deformation occurs with respect to its original position [4]. In classical respiratory physiology, this would correspond to flow divided by FRC.

**Driving pressure (DP).** DP correlates the classic model with the rheological model. DP = plateau pressure − total positive end-expiratory pressure (total PEEP). Marini et al. [6] found a good correlation between stress (PTP) and DP. Chiumello et al. [7] corroborated this point and additionally determined a threshold DP value of 15 cmH_2_O that would have very good diagnostic accuracy for detecting that the lung is being subjected to stress equal to or greater than 12 (strain of 1), the point from which VILI would begin.

**Mechanical power (MP).** In 2016, Gattinoni et al. [8] developed the concept of MP. To date, this is the model that has most successfully approximated rheological theory and classical respiratory physiology. It attempts to combine all parameters, both static and dynamic, that play a role in generating the energy delivered by the ventilator to the lung and airways per unit time.

**Figure 2:**
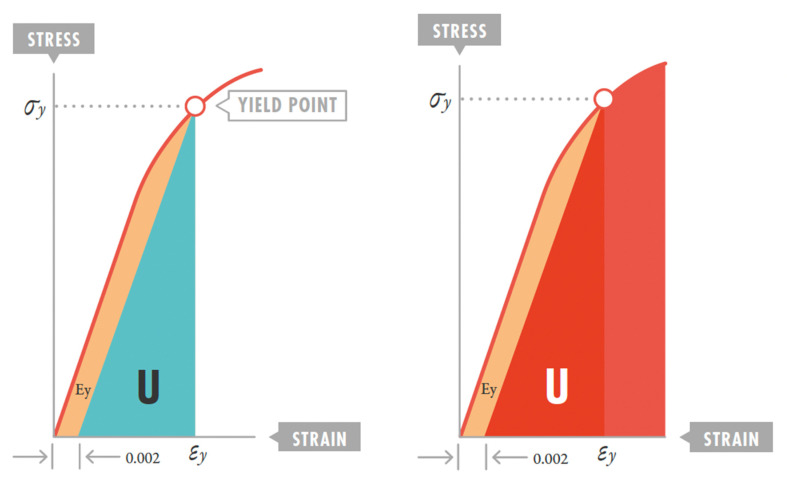
Resilience: maximum energy that can be stored in an elastic body and subsequently recovered without any loss (**left**). Pathogenesis of ventilator-induced lung injury: deformation energy exceeds resilience (**right**). σy: stress at maximum elasticity point; εy: strain at maximum elasticity point; U: resilience. Reproduction with permission: Manual of Pediatric and Neonatal Mechanical Ventilation (SECIP and ESPNIC). 2nd ed. Medina Villanueva A et al. (Eds.). Las Palmas de Gran Canaria (Spain): Tesela Ediciones. 2021.

**Resilience.** This is the deformation per unit volume (J/m^3^) produced within a material when stress is applied from a base state to the yield point [3,4]. It constitutes the maximum capacity of a material to absorb energy when elastically deformed and then, upon unloading, completely recover this energy without losses (Figure 2). Clinically, it defines the safety margin before VILI develops.

**Elastic region:** the portion of the stress–strain curve before the yield point, where deformation is fully reversible. In lung tissue, this represents the safe ventilation zone where mechanical energy can be applied and recovered without permanent structural damage. VILI likely occurs when ventilation pushes the lung beyond this elastic region into plastic deformation territory [4].

By reassessing lung mechanics with these definitions, rheology provides clinically actionable thresholds and offers a better understanding of why conventional strategies sometimes fail.

Barotrauma, volutrauma, atelectrauma, and biotrauma have been reviewed and shaped practice, but they are limited. Evidence from ARDSNet and large meta-analyses established low tidal volume and DP < 15 cmH_2_O as protective. However, recent data show that MP, which integrates pressure, volume, flow, and frequency, is a stronger predictor of VILI. Importantly, preliminary evidence suggests that normalized MP (per kg IBW or per L compliance) better predicts mortality than absolute MP.

## 2. Evolution from Classical to Rheological VILI Models

Since the 1970s, two static variables have been fundamentally studied as VILI inducers: pressure and volume.

Initially, the concept of barotrauma emerged, relating to excessive peak airway pressure (greater than 36 cmH_2_O). A decade later, experimental studies demonstrated that when generated volumes were relatively low, pressures up to 45 cmH_2_O did not induce lung injury, and high volumes did cause injury despite using low pressures [9].

Volume was then considered the main determinant of VILI. This concept continued to develop, and in 2000, the ARDS Network trial was published in *The New England Journal of Medicine* [10], proving that ventilating ARDS patients with volumes of 6 mL/kg ideal body weight versus 12 mL/kg reduced mortality.

Simultaneously with volutrauma studies, several works began investigating the modulating role that PEEP could have. In the 1990s, this gave rise to the concept of atelectrauma, which posits that periodic opening and closing of collapsed alveolar units also generate tissue damage. Marcelo Amato et al. [11] published in 1995 one of the first clinical studies in ARDS patients comparing this new ventilatory strategy, called “open-lung,” with the classical strategy. It underlined the importance of using PEEP above the lower inflection point of the pressure-volume curve.

In the late 1990s, Slutsky et al. [12] developed the concept of biotrauma, which attributes part of both local and systemic lesions to the inflammatory reaction triggered in damaged lung tissue.

In summary, the classical theories explaining VILI development have been:

1. **Barotrauma:** lung injury caused by excessive airway pressures [9,13].
2. **Volutrauma:** damage due to elevated tidal volumes [9,13].
3. **Atelectrauma:** injury from cyclic opening and closing of alveolar units [9,13].
4. **Biotrauma:** inflammatory response secondary to mechanical damage [12].

However, these theories present important limitations:

González-López et al. [14] demonstrated in 2012 that patients ventilated with lower driving pressures (22.6 ± 6 cmH_2_O) but with lower PEEP experienced greater pulmonary inflammation despite receiving protective tidal volumes of 7 mL/kg. This finding directly contradicts barotrauma theory, suggesting that pressure alone does not explain lung damage.

Rahaman [15] provided mathematical proof that ventilation of healthy lungs with tidal volumes of 6–10 mL/kg should not cause VILI, since the generated strain (0.17–0.29) produces stress (2.31–3.86 cmH_2_O) well below the harmful threshold. However, upon reaching total lung volume (strain of 1.5), the resulting stress (17 cmH_2_O) exceeded the safety threshold, explaining why conventional ventilatory strategies do not cause damage in healthy lungs but can be injurious in ARDS.

The LUNG SAFE study [16] and Amato et al.’s meta-analysis [17] in 2015 revealed that DP was the factor most consistently associated with mortality, regardless of tidal volume, plateau pressure, or PEEP values used, thus questioning classical theories.

These findings suggest that traditional theories are incomplete and that a model based on rheological principles offers a more coherent explanation of VILI mechanisms.

Rheological theory considers the lung as a viscoelastic body whose behavior can be described by Voigt’s constitutive equation [18]:

**Stress = E_Y_ × Strain + η × Strain rate**

**E_Y_**: Young’s modulus (specific elastance); **η**: viscosity coefficient

Thus, from a physical standpoint, it is reasonable to assume that the respiratory system behaves as a viscoelastic body. At least three phenomena related to the viscoelastic properties of the ventilated respiratory system have been identified:

Dynamic hysteresis (in the dynamic pressure-volume loop).

Relaxation index or stress relaxation (P1–P2 difference after a 5 s inspiratory pause).

Stress index.

## 3. New VILI Concepts Related to Rheological Theory

### 3.1. The Lung as an Elastic Solid

Gattinoni et al. [19] demonstrated in 2008 and Protti et al. [20] in 2011 that the lung behaves like an elastic solid, with a linear relationship between stress and strain, fulfilling Hooke’s law:

**ΔPTP = ESL × (V_T_/FRC)**

**ΔPTP**: transpulmonary pressure increment; **ESL**: specific lung elastance; **V_T_**: tidal volume; **FRC**: functional residual capacity

Specific lung elastance (**ESL**) in adults with ARDS is remarkably constant: 13.5 ± 4.1 cmH_2_O (95% CI: 11.8–15.2), regardless of ARDS cause, **V_T_**, or PEEP used [19]. In children with ARDS, this value is similar: 13.5 cmH_2_O (95% CI: 10–15.3) [21]. This value does not change with age.

### 3.2. Strain Threshold and VILI Development

The lung’s stress–strain curve shows a linear relationship up to strain values near 1, losing linearity between 1.5 and 2. This point marks the elastic limit beyond which irreversible microfractures begin to occur in lung parenchyma [20].

In animal experiments, Protti et al. [20] demonstrated that VILI risk increased exponentially with strain value. Animals without VILI presented strain values of 1.29 ± 0.57, while those with VILI had values of 2.16 ± 0.58 (*p* < 0.001), with associated mortality of 86% after only 60 h of mechanical ventilation.

### 3.3. Stress and Strain Estimation with the Ventilator

The rheological model and classical elastic-resistive model represent distinct paradigms that were historically considered immeasurable. However, recent studies have demonstrated functional equivalence between both approaches through the use of DP as a bridging parameter. The main limitation to applying the rheological model in clinical practice is the difficulty of estimating FRC at the bedside using a conventional ventilator. However, recent studies have established functional equivalence between both approaches [7].

Cortés-Puentes et al. [6] demonstrated a consistent relationship between rheological stress (ΔPTP) and driving pressure (DP), a parameter easily measurable clinically as the difference between plateau pressure (Pplat) and total PEEP, both measured under static conditions. The ΔPTP/DP ratio oscillates between 0.46 and 0.79, being approximately 0.75 in ARDS patients, and remains stable with PEEP changes, although it decreases with elevated intra-abdominal pressure (except in ARDS).

Chiumello et al. [7] replicated these findings and confirmed a significant correlation between stress and DP (R^2^ = 0.7; *p* < 0.001). In ARDS patients, stress accounts for between 73% and 85% of DP. Considering that lung Young’s modulus (specific elastance) in human ARDS is 13 cmH_2_O, it follows that pulmonary stress equal to or greater than this value can induce VILI.

Chiumello et al. [7] validated DP use as a marker of injurious stress, establishing a clinical threshold of 15 cmH_2_O with high diagnostic capacity (AUC = 0.864; 95% CI: 0.80–0.93; sensitivity = 0.90; specificity = 0.78). This threshold identifies the presence of pulmonary stress ≥ 12 cmH_2_O, associated with VILI risk.

In conclusion, DP represents the key parameter that allows establishing a connection between the classical elastic-resistive model and the rheological model, thus providing functional equivalence between both paradigms. A DP threshold value of 15 cmH_2_O, which in humans is associated with an approximate strain of 1.5, appears to mark the elastic limit beyond which VILI is induced.

Along these lines, Rahaman et al. [15], applying rheological theory, offer a coherent explanation for a widely observed fact in clinical practice: conventional mechanical ventilation does not induce VILI in healthy lungs. Under physiological conditions, healthy lung FRC is approximately 35 mL/kg, while total lung capacity (TLC) reaches 85 mL/kg [22]. If a ventilatory strategy were applied that brought end-inspiratory volume to TLC and end-expiratory volume to FRC, the resulting strain would be 1.5. This deformation level translates, according to specific elastance modulus (ESL), into stress (ΔPTP) equivalent to a driving pressure clearly superior to the 15 cmH_2_O threshold, thus positioning in a range capable of generating VILI even in a previously healthy lung.

Conversely, in the same lung, using tidal volumes of 6 mL/kg (strain = 0.17; stress = 2.31 cmH_2_O; DP = 2.89 cmH_2_O) or even 10 mL/kg (strain = 0.29; stress = 3.86 cmH_2_O; DP = 4.82 cmH_2_O) is considered safe, as it remains well below the injurious threshold. This practice is the same as the ventilatory strategy traditionally employed in patients without lung disease, supporting its safety from a rheological perspective.

### 3.4. Mechanical Power and Injury Threshold

MP integrates all factors capable of producing VILI (V_T_, pressures, frequency, and flow) into a single physical magnitude representing energy transmitted to the respiratory system per unit time [23].

The simplified formula for its calculation is:

**MP (J/min) = 0.098 × RR × V_T_ × (PIP − DP/2)**

**MP**: mechanical power; **RR**: respiratory rate; **V_T_**: tidal volume; **PIP**: peak inspiratory pressure; **DP**: driving pressure.

Gattinoni et al. [8] experimentally demonstrated the existence of an MP threshold (approximately 12 J/min) above which VILI occurs, regardless of the combination of ventilatory parameters used.

While the simplified Gattinoni formula is most widely cited, alternative formulations exist for clinical practice:

1. Giosa et al.’s [24] surrogate method: Proposed a more comprehensive calculation for volume-controlled ventilation, incorporating resistive and elastic components.
  MP = 4 × DP × RR.
  MP: mechanical power; RR: respiratory rate; DP: driving pressure.
2. Becher et al.’s [25] method: Adapted calculations for pressure-controlled ventilation, emphasizing that MP estimates differ by mode.
  MP = 0.098 × RR × VT × [PEEP + (DP × ((Ti/60 × RR) + 1))/2]

MP: mechanical power; RR: respiratory rate; V_T_: tidal volume; PEEP: positive end-expiratory pressure; Ti: inspiratory time; DP: driving pressure.

Each method has specific applications and limitations. These variations illustrate that MP is a concept rather than a single formula, and calculation methods may yield slightly different results in practice. The clinical application of MP remains controversial. Supportive studies [26,27,28] show associations between higher MP and mortality in ARDS. However, debates persist regarding formula choice, threshold generalizability, and individual variability. Therefore, MP should be considered a research concept, with normalization by IBW or compliance being an emerging refinement that requires further validation.

## 4. Ventilatory Strategy in ARDS from a Rheological Perspective

The following ventilatory strategy recommendations integrate rheological principles with existing clinical evidence. Recommendations are graded based on the quality of supporting evidence, with a clear distinction between established practices and emerging concepts requiring further validation.

### 4.1. Mechanical Power Adjustment by Ideal Weight or Compliance

MP adjustment by ideal body weight (IBW) or compliance (C) is a crucial aspect of the rheological model that deserves greater attention since MP as an absolute value (12 J/min) presents important limitations by not considering individual patient characteristics [8,23,27,28,29,30,31]:

1. It was derived primarily from studies in animal models with homogeneous anatomy/structural features.
2. Body size differences between patients were not accounted for.
3. Differences in the proportion of functional “baby lung” available in each ARDS case were not adjusted.
4. Differences in lung compliance between patients were not factored in.

Thus, MP could be normalized by the patient’s ideal weight [27,28,29,30,31], providing a more individualized value:

- **Normalized MP (J/min/kg) = Total MP (J/min)/Ideal weight (kg).**

Preliminary studies suggest that a threshold of approximately 0.25–0.3 J/min/kg might be more appropriate.

This approach is similar to V_T_ normalization by ideal weight already routinely used.

Or it could be normalized by compliance [27,28,29,30,31], potentially more precise than ideal weight adjustment, as it directly reflects functional lung tissue:

- **Specific MP (J/min/L) = Total MP (J/min)/Static compliance (L/cmH_2_O)**

The advantage of this approach is that it considers the severity of lung involvement in each individual patient, adjusting the “dose” of mechanical energy according to available functional tissue. This adjustment attempts to represent the theory of ARDS lung as a “baby lung” [32].

Some recent studies have begun exploring these normalizations (IBW and compliance):

Serpa Neto et al. [26] found that compliance-normalized MP showed better correlation with mortality than absolute MP.

Gattinoni et al. [32] subsequently proposed the concept of “specific mechanical power” that considers the baby lung.

Cressoni et al. [23] suggested that the injury threshold might be between 0.12 and 0.15 J/min per mL of functional lung tissue.

Ito et al. [29] have shown that weight adjustment underestimates MP compared to compliance-adjusted MP in pediatrics.

MP normalization would have important practical implications (Table 2) [24,25,27,30,31,33,34]:

1. Patients with severe ARDS (lower compliance) would require lower absolute MP thresholds.
2. Patients with preserved compliance could tolerate higher absolute MP values.
3. Ventilatory strategy could be dynamically adjusted according to compliance evolution.
4. It would allow more precise comparisons between patients with different characteristics.

However, implementing this approach requires the following:

1. Clinical studies validating specific normalized MP thresholds.
2. Practical methods to estimate baby lung volume at bedside.
3. Algorithm development to automatically adjust ventilatory parameters according to normalized MP.
4. Validation in specific populations (pediatric, obese, etc.).

In summary, this perspective on MP adjustment by ideal weight or compliance represents an important advancement in individualized protective ventilation and aligns with the fundamental principles of the rheological model. However, it is important to note that research in this field continues evolving, and more studies are needed to establish definitive normalized MP thresholds.

### 4.2. VILI Development Dynamics and Recruitment. Optimal PEEP

VILI is a time-dependent phenomenon. Gattinoni et al. [35] demonstrated in 2013 that VILI development follows a temporal relationship with applied strain:

**With a strain of 2.5:** detectable VILI occurs at 6 h with 100% mortality at 48 h.

**With a strain of 1.154 and 0.556:** VILI was detectable at 24 and 36 h, respectively.

**With a strain of 0.217:** absence of VILI after 60 h of ventilation despite using end-inspiratory volumes equal to TLC (with initial inspiratory Pplat values of 36 ± 3 cmH_2_O), not only does VILI not appear, but Pplat values at experiment end were the lowest among the four cohorts.

It is worth noting that none of the theories that attempt to explain the etiopathogenic mechanism of VILI (barotrauma, volutrauma, atelectrauma, biotrauma) to date have explicitly considered a mechanism that includes ventilation time as an important factor.

Therefore, VILI development occurs exponentially, initiating in interface zones of naturally non-homogeneous structures (subpleural microatelectasiae, peribronchial and intraparenchymal areas). These zones act as “stress multipliers” (stress raisers), similar to material fatigue mechanisms [1].

A crucial finding was that these initial VILI densities only appear in computed tomography images performed during expiration, indicating they are highly recruitable areas. This suggests that adequate PEEP use could prevent their appearance by maintaining more homogeneous parenchyma [36].

Protti et al. [18] observed that both low and high strain rate groups showed lower stress relaxation (P1-P2 difference) when ventilated with 10 cmH_2_O PEEP compared to 0 cmH_2_O PEEP (ZEEP). This effect was independent of strain rate, suggesting that PEEP reduces the viscous behavior of lung parenchyma during ventilation.

The use of sufficiently elevated PEEP level (so-called optimal PEEP) is the most relevant parameter in lung-protective strategy. It has a very important effect (NNT = 5 patients; 95% CI = 3 to 10) in reducing mortality in severe ARDS patients [37]. Maintaining permanent opening of the highest possible percentage of recruitable alveoli through high continuous distending pressure prevents VILI because it prevents atelectrauma caused by cyclic collapse of these alveoli [38]. To correctly apply the open-lung strategy [11,39,40,41], high PEEP must be optimized according to each patient’s lung mechanics. For this, it is necessary that all patients have their static compliance curve analyzed in its inspiratory ramp (for example, through the “super-syringe” technique), prescribing a PEEP level 2 cmH_2_O above the lower inflection point (LIP) of this curve. This is the transpulmonary pressure point corresponding to that patient’s “functional residual capacity (FRC).” With this PEEP level, the entire respiratory cycle happens above FRC, just as in healthy lungs. Atelectrauma is avoided, and intrapulmonary shunt is minimized (therefore, FiO_2_ requirements are reduced).

Data from open lung strategy clinical trials makes it possible to infer that this LIP is situated in 95% of cases between 12.35 and 13.43 cmH_2_O. Therefore, to be 97.5% certain that the patient receives optimal PEEP, approximately 15 cmH_2_O PEEP should be prescribed.

Based on statistical analysis of open-lung strategy trials, the lower inflection point is in 95% of cases between 12.35 and 13.43 cmH_2_O, suggesting that PEEP levels around 15 cmH_2_O may provide optimal recruitment in the majority of ARDS patients. However, this represents a population-derived estimate that requires individualization based on:

Critical considerations:

- Hemodynamic tolerance assessment.
- Individual recruitability testing (P/F ratio response).
- Chest wall mechanics and intra-abdominal pressure.
- Underlying cardiac function.
- Real-time monitoring of compliance and driving pressure.

The 15 cmH_2_O level should serve as a starting point for PEEP titration rather than a universal target. Some patients may require higher PEEP for optimal recruitment, while others may not tolerate this level due to hemodynamic compromise or limited recruitability.

This achieves a partial arterial oxygen pressure/inspired oxygen fraction (P/F) ratio between 170 and 200, indicating that shunt will have been reduced to 30–40%. Higher PEEP levels may be necessary, sometimes requiring Pplat values above 32 cmH_2_O, provided the 15 cmH_2_O limit in DP is not exceeded.

Generating a static compliance curve at the bedside is usually not feasible, and therefore the “optimal” PEEP level must be determined indirectly. Kacmarek, Amato, and other investigators [42] have verified that determining PEEP level based on the static compliance curve LIP and performing so by measuring the shunt degree produces results without statistically significant differences.

Therefore, in the clinical setting, it seems reasonable to determine “optimal” PEEP level based on the P/F ratio value obtained with it [43]. A P/F ratio > 150–175 seems a reasonable objective. Clinical trials using higher PEEP levels (super PEEP trials) have only managed to demonstrate very marginal survival improvement [44,45,46], particularly in the most severe patients [47,48]. Any further PEEP increase will result in a merely “cosmetic” effect and subject the patient to unnecessary hemodynamic compromise. Recently, it has been described that daily lung mechanics trends can be assessed based on real-time responses of respiratory system compliance, end-expiratory lung volume, and stress and strain with respect to PEEP changes using a non-invasive nitrogen washout technique [31]. There is now evidence that, although a PEEP increase up to 10 cmH_2_O increases tension and stress, both remain below known harmful values in pediatric ARDS [33].

In summary, the hypothesis is that PEEP’s effect on VILI incidence, from a rheological perspective, relates to its capacity to:

**Increase FRC:** by increasing the denominator in the strain equation (V_T_/FRC), it reduces strain for the same V_T_.

**Homogenize lung parenchyma:** it prevents “stress multiplier” formation by maintaining recruited unstable alveolar units.

However, it is important to highlight that PEEP does not always increase FRC in ARDS patients, and its effect on lung recruitment is difficult to predict at bedside.

To the present day, the PEEP effect on VILI production has not yet been fully established experimentally within the new rheological theory framework.

### 4.3. Importance of Respiratory Rate

Respiratory rate (RR) plays a fundamental role in VILI development. Marini et al. [49] first described in 2000 that elevated RR worsened VILI in an animal model.

Karolinska Institute researchers [50] stated, using a “double hit” model (alveolar lavage followed by aggressive ventilation), that animals ventilated with 40 bpm RR presented worse P/F ratio and higher biochemical and anatomopathological VILI markers compared to those ventilated at 20 bpm, despite using protective strategies (6 mL/kg V_T_, 10 cmH_2_O PEEP).

This effect is explained by RR’s contribution to total mechanical power:

**MP = RR × V_T_ × Pressures**

**MP**: mechanical power; **RR**: respiratory rate; **V_T_**: tidal volume

Elevated RR linearly increases MP delivered to the respiratory system, potentially exceeding the safety threshold even with protective tidal volumes.

### 4.4. Importance of Flow

Gattinoni’s group [51] described that not only is strain magnitude important, but also the velocity at which it occurs (strain rate). In experiments with pigs ventilated with a similar strain (2.1 ± 0.9) but different flow velocities:

The high strain rate group (I:E ratios of 1:5 to 1:9) presented:

Worse compliance and P/F.

Higher inflammatory markers and anatomopathological signs of pulmonary edema.

Higher VILI prevalence (73% vs. 20%, *p* = 0.01).

Higher mortality at 54 h (47% vs. 13%).

These findings suggest that high inspiratory flows increase VILI risk by increasing strain rate, even when maintaining other ventilatory parameters constant.

Additionally, the group with greater VILI presented higher values in dynamic pulmonary hysteresis index (in Joules) and stress relaxation index (P1–P2, cmH_2_O), parameters that can be monitored in clinical practice.

**Figure 3:**
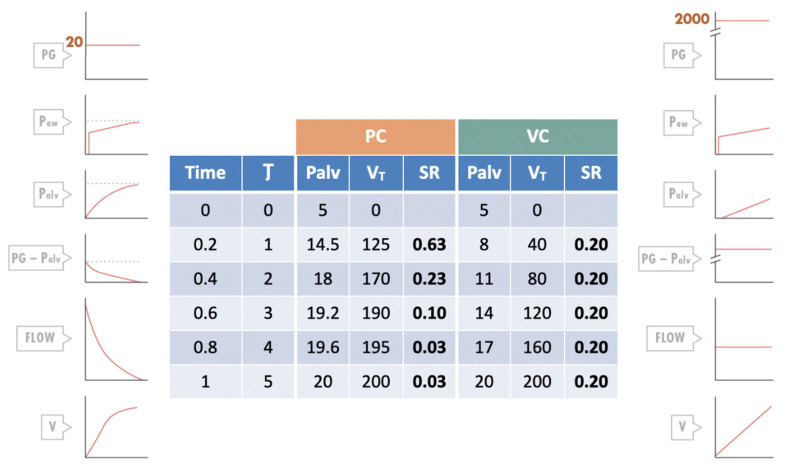
Comparative example of gas delivery in pressure control (PC) and volume control (VC). Time constant (τ) is considered to be 0.2 s (5 time constants are completed in 1 s). Ideal weight is 30 kg and age 8 years. The estimated functional residual capacity is 1000 mL. Tidal volume (V_T_) to be delivered is 200 mL, and plateau pressure (Pplat) or alveolar pressure (Palv) to be reached when gas delivery is completed is 20 cmH_2_O. τ: time constant; PG: generator pressure (working pressure); Paw: airway pressure; Palv: alveolar pressure; FLOW: airflow (L/min); V: volume; SR: strain rate. Reproduction with permission: Manual of Pediatric and Neonatal Mechanical Ventilation (SECIP and ESPNIC). 2nd ed. Medina Villanueva A et al. (Eds.). Las Palmas de Gran Canaria (Spain): Tesela Ediciones. 2021.

To illustrate the gas delivery problem, an example can be used comparing VC and PC (Figure 3). Despite the strain being the same, the strain rate will be three times higher in PC in the first time constant.

### 4.5. Importance of Ventilatory Mode

Ventilatory mode choice significantly influences VILI development, mainly through its effect on flow pattern.

In a series of experiments [52,53,54] comparing continuous mandatory pressure-controlled modes with an adaptive control scheme (PC-CMVa) versus continuous mandatory volume-controlled modes with a fixed control scheme (VC-CMVs), VC-CMV modes were clearly superior in both oxygenation parameters and anatomopathological findings. That is, PC-CMVa modes produced more VILI.

This surprising finding may be related to the higher peak flow necessary in PC-CMVa to introduce the same volume (same strain), inducing a higher strain rate, as Gattinoni’s team has proved [51].

Schmidt et al. [55] have also demonstrated that ventilation with expiratory flow control attenuates lung injury in a porcine ARDS model. This suggests that not only is inspiratory flow important, but also deformation velocity during expiration (expiratory strain rate).

High-frequency oscillatory ventilation (HFOV) use represents an illustrative example of this principle. According to traditional volutrauma/barotrauma theories, HFOV should be safer than conventional mechanical ventilation since it delivers V_T_ below anatomical dead space with moderate continuous distending pressure.

However, the available literature has not shown its superiority, and worse outcomes have been reported when using HFOV compared to conventional mechanical ventilation OSCILLATE and OSCAR trials [56,57]. The rheological model can explain this apparent paradox: HFOV uses simple harmonic motion that transmits significant energy to lung tissue, potentially exceeding mechanical power thresholds despite low V_T_. Important confusing factors: the negative HFOV trial results may also be influenced by the following:

- Late initiation timing (after conventional ventilation failure).
- Patient selection bias (most severe cases).
- Suboptimal HFOV settings in some centers.
- Learning curve effects in participating institutions.

These factors highlight the complexity of translating rheological principles into clinical practice and the need for careful consideration of multiple variables beyond purely mechanical power calculations.

### 4.6. Tidal Volume and Driving Pressure

In all mammalian species [58], physiological V_T_ is 6.3 mL/kg. The V_T_ limitation to 6 mL/kg constitutes one of the fundamental aspects of lung-protective ventilation strategy. Lung involvement in ARDS is very heterogeneous, with collapsed non-recruitable zones coexisting with healthy or recruitable zones that remain open throughout the respiratory cycle. If very high V_T_ values are used, these ventilatable zones receive all delivered air (the volume that should come to them plus the remaining that should go to collapsed zones). V_T_ limitation aims to avoid overdistention of these open zones. It is a therapeutic measure that, although not proven to decrease barotrauma, has managed to moderately decrease ARDS patient mortality [10] (number needed to treat [NNT] = 12 patients; 95% CI: 8 to 36). But it is essential that this V_T_ limitation be accompanied by adequate PEEP to maximize recruitment and decrease overdistention by distributing air among the highest possible number of alveoli.

Since the beginnings of lung protective ventilation [59], V_T_ limitation was established indirectly by limiting maximum Pplat (35 cmH_2_O) in pressure control modes. This maximum Pplat correlates with the PTP needed to insufflate lungs of a paralyzed and ventilated adult to obtain TLC [60]. However, experimental animal models have helped describe that the main cause of overdistention is applied V_T_ rather than maximum Pplat [9]. This implies that absence of V_T_ limitation can cause VILI despite Pplat < 30 cmH_2_O and, conversely, lower V_T_ (6 mL/kg) using sufficient PEEP and recruitment maneuvers could make it possible for the patient to tolerate higher Pplat (40 cmH_2_O) [45]. Although this topic remains controversial, subsequent consensus guidelines for ARDS ventilatory management, for both adults and children, established a maximum Pplat limit of around 30 cmH_2_O [61,62,63].

In relation to this, DP has been independently associated with mortality. Amato’s study [17] states that Pplat increase is not always deleterious nor PEEP increase always protective, but rather the pressure difference between both that causes lung injury. A differential pressure below 15 cmH_2_O has been established as a safety point [17].

On the other hand, there is no evidence that using less than 6 mL/kg V_T_ is positive for ARDS patients. In fact, since recruitment occurs through insufflation pressure (and is maintained by PEEP), using lower V_T_ could compromise potential recruitment in some lung regions, thereby worsening atelectrauma. In neonates with hyaline membrane disease, it has been observed that using 3 mL/kg V_T_ versus 5 mL/kg produces greater pulmonary inflammation [64]. Consistent with this, Eichacker et al. [65] published meta-analysis data indicating that V_T_ < 5 mL/kg should not be used routinely since both high and low V_T_ use can increase mortality. With Pplat of 28–32 cmH_2_O being what most decreases mortality. This point has also been highlighted by the Cochrane meta-analysis published in 2013, which emphasizes that mortality does not increase until Pplat is greater than 32 cmH_2_O [66].

Based on all the above and supported by Costa et al. 2021 meta-analysis [28], from a practical standpoint, initial V_T_ should be 6 mL/kg ideal weight. If, having programmed adequate initial PEEP (15 cmH_2_O), Pplat is equal to or greater than 32 cmH_2_O, then V_T_ should be decreased, with 4.5 mL/kg ideal weight recommended as the lower limit.

Classically, in cases where V_T_ is below 6 mL/kg, minute volume loss was compensated with RR increase. However, the resulting acidosis from the permissive hypercapnia-based strategy is less injurious than the V_T_ increase necessary to correct it. And if accompanied by other lung protection measures (especially adequate PEEP level [67], this can help improve hemodynamics of adult patients ventilated for ARDS [68].

This supposed protective effect of permissive hypercapnia cannot be explained by classical VILI theories, but it may align with the new vision provided by rheological theory. Considering that RR has a direct effect on MP increase, this can explain the beneficial effect of hypercapnia.

### 4.7. Resilience Implications in ARDS Ventilatory Strategy

Material resilience is deformation per unit volume (J/m^3^) produced within a material by deformation when stress is applied from a base state to the yield point. It constitutes the maximum capacity of a material to absorb energy when elastically deformed and then, upon unloading, completely recover this energy without losses. Since the first part of the stress–strain curve is linear, resilience is calculated as the geometric area of the triangle below the initial linear elastic region of that curve, up to the yield point (Figure 2). Therefore, it is half the product of stress and strain within that first segment [3,14,35].

When deformation energy remains within this elastic “comfort” zone, there is no energy loss. Then, there is no excess energy that leaks into the material, producing microfractures and deformations, ending with material rupture (yield limit) (Figure 2) [8,23,69].

This threshold effect is a prediction of materials science. It requires staying in the deformation “comfort” zone to avoid creating microfractures. Therefore, to minimize VILI, it is important to adjust ventilator settings (in non-invasive and invasive ventilation) to maintain the energy level involved so that it is less than human lung resilience (J/m^3^), diseased or not. If deformation energy exceeds the safe zone, VILI appears [3].

#### Clinical Implications of Resilience

1. Safe energy threshold:

Defined therapeutic window: Resilience establishes a quantifiable energy limit for safe ventilation. The energy delivered to the respiratory system must be maintained below this threshold to prevent irreversible microfractures [8,70].

Assessment of ventilatory strategies: Any combination of ventilatory parameters (volume, pressure, flow, frequency) must be evaluated in terms of the total energy delivered in relation to the patient’s pulmonary resilience [30,69].

2. Personalization of mechanical ventilation:

Dynamic adjustment: Resilience may vary between patients and within the same patient over time. This justifies the need for continuous monitoring and adjustment of ventilatory parameters [14,19].

Functional reserve assessment: Resilience provides information about the respiratory system’s “reserve” before entering the irreversible deformation zone, allowing ventilation optimization according to individual capacity [35,71].

3. VILI prevention:

Unifying pathophysiological mechanism: Resilience offers an integrative explanation for why different combinations of ventilatory parameters can produce VILI when total energy exceeds this threshold [8,69].

Energy redistribution: Maintaining ventilation within resilience limits involves appropriately distributing energy among all parameters (reducing volume, frequency, or flow when one must be increased) [51,70].

4. PEEP optimization:

PEEP as resilience modulator: Optimal PEEP homogenizes lung parenchyma, potentially increasing global resilience by reducing “stress multipliers” and more uniformly distributing applied energy [43,50].

Balance between recruitment and overdistension: PEEP should be adjusted to maximize lung tissue operating within its resilience zone, avoiding both cyclic collapse and overdistension [17,19].

5. Permissive hypercapnia management:

Physiological rationale: Permissive hypercapnia finds support in the resilience concept, as it prioritizes maintaining delivered energy within safe limits even at the cost of suboptimal minute ventilation [72,73].

Energy-based clinical decisions: The decision to permit hypercapnia can be based on maintaining energy below the resilience threshold when increasing minute ventilation would exceed it [30,69].

6. Prone position ventilation:

Stress and strain redistribution: Prone positioning can homogenize force distribution in lung parenchyma, allowing a greater proportion of tissue to operate within its resilience zone [19,35].

Energy optimization: Prone positioning could allow for the delivery of the same total energy with reduced VILI risk by improving regional resilience distribution [14,50].

7. Monitoring and biomarkers:

Resilience threshold breach markers: Parameters such as dynamic hysteresis index and stress relaxation index (P_1_–P_2_) could serve as indicators that pulmonary resilience capacity is being exceeded [35,74].

Development of new tools: The concept justifies the development of monitoring systems that assess in real-time whether ventilation remains within resilience limits [71,74].

8. Non-invasive ventilation applications:

Risk assessment: The resilience concept can be applied to evaluate P-SILI (patient self-induced lung injury) risk during NIV, especially in patients with vigorous respiratory efforts [73,75].

Intubation criteria: The inability to maintain breathing within resilience limits could constitute an objective criterion for deciding intubation in patients receiving non-invasive support [30,71].

**Practical application:**

In daily clinical practice, the resilience concept translates to:

1. Maintaining driving pressure below 15 cmH_2_O [17].
2. Limiting total mechanical power to less than 12 J/min (ideally normalized by weight or compliance) [8,70].
3. Reducing respiratory rate to the minimum necessary for adequate ventilation [50].
4. Using flow patterns that minimize strain rate [51,55].
5. Implementing optimal PEEP to homogenize parenchyma [43].
6. Considering deep sedation and muscle relaxation in the initial phases to prevent patient respiratory effort from contributing to resilience threshold breach [73,75].

The resilience concept thus provides a solid theoretical framework that unifies multiple aspects of protective ventilation, enabling advancement toward more personalized strategies based on fundamental physical principles [30,69].

### 4.8. Self-Inflicted Lung Injury (SILI)

Classically, preserving patients’ spontaneous breaths was recommended; in fact, this is the basis for justifying Airway Pressure Release Ventilation (APRV) use. However, it has been demonstrated that muscle relaxant use in the most acute phase after intubation decreases mortality [76]. This data has been confirmed in a meta-analysis [77].

The concept of self-inflicted lung injury extends the rheological model to patients with preserved spontaneous breathing effort [30,73]. During vigorous respiratory efforts, particularly in ARDS, elevated negative pleural pressures can produce lung injury through similar mechanisms as mechanical ventilation.

Pathophysiological mechanisms:

**Increased regional strain.** Especially in dependent zones, where negative pleural pressure can cause excessive local deformations.

**Pendelluft phenomenon.** Air movement between lung regions with different time constants, increasing regional strain without changes in total V_T_.

**Increased strain rate.** Vigorous inspiratory efforts generate high flows that increase deformation velocity.

**Increased mechanical power (ventilator + patient effort).** The sum of the patient’s respiratory work and energy delivered by the ventilator can exceed the lung resilience threshold.

From a rheological perspective, SILI can be prevented through the following:

Prevention strategies (evidence-graded):

High-quality evidence:

- Neuromuscular blockade in early severe ARDS (strong recommendation, high-quality evidence):
  - Based on the ACURASYS trial [76] and systematic reviews [77].
  - NNT = 8 for mortality benefit in severe ARDS.
  - Duration: 48 h in the first week of ARDS.

Moderate-quality evidence:

- Adequate sedation to minimize excessive respiratory drive (conditional recommendation, moderate-quality evidence).
  - Reduces metabolic demand and respiratory effort.
  - Must balance with delirium prevention.

Expert consensus/low-quality evidence:

- Ventilatory mode selection to minimize patient-ventilator asynchrony (Expert consensus).
- Esophageal pressure monitoring in selected cases (Expert consensus).
- Early consideration of ECMO in refractory cases (Conditional recommendation, Low-quality evidence).

Important limitation: Much of SILI prevention remains based on pathophysiological reasoning and expert consensus rather than high-quality randomized controlled trials. The field requires dedicated studies specifically designed to test SILI prevention strategies.

## 5. Rheological Model Limitations

Some limitations of the new rheological theory are shown below:

### 5.1. Regional Lung Variability Not Captured by the Model

Current mathematical models of lung mechanics have important limitations in capturing regional lung heterogeneity, particularly in pathological states such as ARDS. This marked heterogeneity (areas of alveolar collapse, consolidation, and relatively preserved zones coexisting in the same lung) generates regional mechanical behaviors that global parameters cannot adequately represent. Current models can lead to erroneous interpretations of global lung mechanics by assuming lung homogeneity. ARDS creates ‘stress raisers’ in interface zones between healthy and damaged lung tissue, similar to crack propagation in materials science. These local stress concentrations cannot be captured by global parameters but may be critical for VILI initiation; experimental evidence from Cressoni et al. [23].

### 5.2. Interaction Between Mechanical Ventilation and Inflammation Not Completely Explained

Current models focus primarily on mechanical parameters, without adequately incorporating biological aspects of ventilator-lung interaction. Mechanical ventilation can induce or exacerbate pulmonary and systemic inflammatory responses through mechanisms like biotrauma and mechanotransduction, processes that are not adequately integrated into conventional mechanical models.

Dynamic inflammation-mechanics interaction in mechanical ventilation activates mechanosensitive pathways (integrin signaling, NF-κB activation) that amplify inflammatory responses, creating a positive feedback loop not incorporated in current models. Experimental studies by Chiumello et al. [74] have described how specific ventilation patterns can modulate gene expression and inflammatory mediator release, even in the absence of evident changes in mechanical parameters. This bidirectional interaction between mechanics and inflammation represents a significant challenge for current models.

### 5.3. Challenges for Determining the “Baby Lung” Precisely at the Patient Bedside

The “baby lung” concept, introduced by Gattinoni [32], has been essential to the understanding of ARDS pathophysiology and the adaptation of ventilatory strategies. However, precise quantification of functional lung tissue at the bedside remains a significant challenge. FRC estimation in ARDS is highly variable and dynamic, making compliance-based MP normalization challenging to implement reliably at bedside.

Currently, “baby lung” volume estimation requires advanced imaging techniques like quantitative computed tomography, which is not routinely available in intensive care units. Indirect methods, such as compliance-based or physiological dead space estimation, present important limitations and may not accurately reflect the lung tissue truly available for ventilation.

In relation to these limitations, several solutions are proposed:

Implementation of multicompartmental models incorporating different time constants for different lung regions.

Integration of real-time functional imaging data to adjust regional model parameters.

Development of finite element-based computational simulations considering patient-specific geometry.

Development of integrated models incorporating real-time biological VILI parameters along with mechanical variables to adjust model parameters.

Creation of experimental models allowing characterization of the relationship between mechanical parameters and their specific biological response.

Validation of simplified methods to estimate “baby lung” based on lung mechanics parameters accessible at bedside.

## 6. Conclusions

In summary, the rheological description above explains how the lung exhibits both viscous and elastic behaviors, with the dominance of viscous components being context-dependent when subjected to rapid deformations, resulting in increased energy dissipation as heat and structural damage.

Therefore, the rheological model offers a promising theoretical framework for understanding VILI mechanisms in ARDS, integrating concepts from materials science and thermodynamics. This approach explains why apparently safe ventilatory strategies can cause lung damage, and why interventions like limiting DP, reducing RR, or controlling flow are protective (Table 3). While specific numeric thresholds (DP < 15 cmH_2_O, MP < 12 J/min) offer clinical guidance, these represent population-derived cutoffs that require individualization based on patient characteristics, particularly compliance, body habitus, and ARDS severity. Inter-patient variability is emphasized, and the need for individualized thresholds, especially in pediatrics, obese patients, and those with low compliance.

MP normalization by compliance or ideal body weight represents an emerging refinement that warrants prospective validation. Clinical implementation should proceed cautiously, with these thresholds serving as starting points rather than absolute targets.

Clinical application of these concepts suggests that:

1. DP should be maintained below 15 cmH_2_O to avoid exceeding the lung elastic limit.
2. Total MP should be limited to less than 12 J/min, adjusting tidal volume, respiratory rate, and flow.
3. PEEP should be optimized to homogenize parenchyma and prevent stress multiplier formation.
4. Inspiratory and expiratory flow control can reduce strain rate and minimize the viscous component of lung damage.
5. In patients with spontaneous breathing, the additional effect of respiratory effort on total mechanical power should be considered, justifying muscle relaxant use in the most severe phases of disease.

Finally, this model offers a more solid scientific basis for developing personalized ventilatory strategies and could guide future research on ARDS prevention and treatment.

## Figures and Tables

**Table 1:** Fundamental concepts of rheology applied to the respiratory system.

Concept	Physical Definition	Pulmonary Application	Units
Stress	Force per unit area (f/A)	Transpulmonary pressure (PTP)	cmH_2_O
Strain	Relative deformation (dX − dX_0_)/dX_0_	Tidal volume/Functional residual capacity (V_T_/FRC)	Dimensionless
Strain rate	Deformation velocity	Flow/FRC	s^−1^
Young’s Modulus (E_Y_)	Proportionality constant between stress and strain	Specific lung elastance (ESL)	cmH_2_O
Driving pressure (DP)	Difference between plateau pressure and PEEP	Clinical approximation to pulmonary stress	cmH_2_O
Mechanical power (MP)	Energy per unit time	Energy delivered to the respiratory system per minute	J/min
Resilience	Maximum energy storable without permanent deformation	Energy threshold to prevent VILI	J/m^3^

f: force; A: area; PTP: transpulmonary pressure; cmH_2_O: centimeters of water; dX: the final lung volume; dX_0_: the baseline lung volume (functional residual capacity); V_T_: tidal volume; FRC: functional residual capacity; s^−1^: reciprocal seconds; E_Y_: Young’s modulus; DP: driving pressure; ESL: specific lung elastance; MP: mechanical power; VILI: ventilator-induced lung injury. PEEP: positive end-expiratory pressure; J/min: joules per minute; J/m³: joules per cubic meter.

**Table 2:** Key clinical implications for mechanical power.

Key Clinical Implications: Mechanical Power (MP) Normalization
Traditional approach Absolute MP threshold: 12 J/min (derived from animal studies).│ │• Limitation: Does not account for patient size or lung function.
Proposed normalizations: 1. By ideal body weight (IBW) normalization: - Formula: normalized MP = total MP/IBW (kg). - Proposed threshold: 0.25–0.3 J/min/kg. Example: - 70 kg IBW adult patient→12 J/min/kg = 0.17 J/min/kg (potentially safe). - 30 kg IBW child patient→12 J/min/kg = 0.4 J/min/kg (potentially harmful). 2. By static compliance (more precise): - Formula: specific MP = total MP/static compliance (mL/cmH_2_O). - Proposed threshold: 0.12–0.15 J/min per mL functional lung. Example: - Mild ARDS (Compliance 40 mL/cmH_2_O)→12 J/min/40 = 0.3 J/min/mL. - Severe ARDS (Compliance = 20 mL/H_2_O)→12 J/min/20 = 0.6 J/min/mL.
Clinical impact: Severe ARDS (low compliance) patients may require absolute MP < 6–8 J/min: lower absolute MP needed. Mild ARDS (preserved compliance): higher absolute MP tolerated. Patients with preserved compliance may tolerate MP > 12 J/min. Dynamic adjustment based on compliance evolution during treatment.
Evidence level: Preliminary. Requires prospective validation.

MP: Mechanical power; J/min: joules per minute; IBW: index body weight; Kg: kilograms; cmH_2_0: centimeters of water; mL: milliliters.

**Table 3:** Ventilatory strategy based on rheological model to prevent ventilator-induced lung injury (VILI) in acute respiratory distress syndrome (ARDS).

Parameter	Recommendation	Rheological Justification
Driving pressure (DP)	<15 cmH_2_O	Maintains strain < 1 (elastic limit)
Tidal volume	Adjusted for DP < 15 cmH_2_O Adjusted for Pplat = 28–32 cmH_2_O	Limits stress and strain
PEEP	PEEP titration to maximize homogeneity and recover pulmonary FRC	Reduces stress multipliers Reduces strain Reduces strain rate
Respiratory rate	Lowest possible allowing adequate ventilation	Limits mechanical power
Inspiratory flow	Moderate, avoiding high peaks	Reduces strain rate
Mechanical power	<12 J/min	Below the injury threshold
Inspiratory time	Prolonged (lower flow)	Reduces strain rate
Flow pattern	Constant and square	Optimizes stress distribution Decreases the strain rate
Expiratory flow control	Consider if available	Reduces expiratory strain rate

FRC: functional residual capacity; Pplat: plateau pressure; PEEP: positive end-expiratory pressure; J: Joules; cmH_2_O: centimeters of water; J/min: joules per minute.
